# Integrating Serological and Molecular Data to Characterize Fowl Adenovirus Associated With Inclusion Body Hepatitis in Broiler Chickens From Malaysia

**DOI:** 10.1002/vms3.71033

**Published:** 2026-06-23

**Authors:** Md. Safiul Alam Bhuiyan, Babu Kanti Nath, Lum Mok Sam, Krishnan Nair Balakrishnan, Suman Das Gupta, Subir Sarker

**Affiliations:** ^1^ Faculty of Sustainable Agriculture Universiti Malaysia Sabah Sandakan Sabah Malaysia; ^2^ Biosecurity, Gulbali Institute School of Agricultural Environmental and Veterinary Sciences Charles Sturt University Wagga Wagga New South Wales Australia; ^3^ Biotechnology Research Institute Universiti Malaysia Sabah Kota Kinabalu Sabah Malaysia; ^4^ School of Agricultural Environmental and Veterinary Sciences Faculty of Science and Health Charles Sturt University Wagga Wagga New South Wales Australia; ^5^ Biomedical Sciences & Molecular Biology College of Medicine and Dentistry James Cook University Townsville Queensland Australia; ^6^ Department of Microbiology Anatomy, Physiology and Pharmacology School of Agriculture Biomedicine and Environment La Trobe University Melbourne Victoria Australia

**Keywords:** broiler chickens, enzyme‐linked immunosorbent assay (ELISA), *hexon* gene, inclusion body hepatitis (IBH), molecular characterization

## Abstract

**Background:**

Inclusion body hepatitis (IBH), caused by fowl adenoviruses (FAdVs), is an emerging disease of commercial broilers associated with significant economic losses. In Malaysia, molecular epidemiological data on circulating FAdV serotypes have largely been restricted to Peninsular regions, with limited information available from East Malaysia (Sabah), despite its rapidly expanding poultry industry and distinct production systems. This regional knowledge gap limits comprehensive understanding of FAdV transmission dynamics at the national level.

**Objectives:**

This study aimed to detect FAdV infection in broiler flocks with IBH‐compatible lesions in Sabah, Malaysia, and to characterize circulating serotypes and their phylogenetic relationships.

**Methods:**

Thirty pooled tissue samples (liver and gizzard) and 60 serum samples were collected from three broiler farms (YAN, KON, and NIS). FAdV detection was performed using polymerase chain reaction (PCR) targeting the *hexon* gene. Serological responses were assessed using enzyme‐linked immunosorbent assay (ELISA). Positive PCR products were sequenced, and phylogenetic analysis was conducted to determine serotype distribution and genetic relatedness.

**Results:**

FAdV DNA was detected in 33.3% (10/30; 95% CI: 17.3–52.8) of pooled tissue samples, with detection rates of 70% (95% CI: 44.4–97.5), 20% (95% CI: 2.5–55.6) and 10% (95% CI: 0.3–44.5) in the YAN, KON and NIS farms, respectively. Although descriptive differences were observed among farms, these variations were not statistically significant (*p *> 0.05). All positive samples yielded the expected 897 bp *hexon* gene amplicon. ELISA revealed high seropositivity in unvaccinated flocks, with the highest mean antibody titre observed in the YAN farm (24,716.5 ± 4516.1). Molecular characterization identified FAdV‐8b (species E) and FAdV‐11 (species D), indicating co‐circulation. Phylogenetic analysis showed close relatedness to strains from Korea, Belgium, China and Australia.

**Conclusions:**

The co‐circulation of FAdV‐8b and FAdV‐11 in Sabah broiler farms underscores the need for continuous molecular surveillance, enhanced biosecurity and breeder‐level vaccination strategies using locally circulating FAdV strains to control IBH in Malaysia.

## Graphical abstract

This study revealed the active circulation of fowl adenoviruses in broiler farms in Sabah, Malaysia. FAdV‐8b and FAdV‐11 were identified in flocks with IBH lesions, accompanied by high antibody titres in unvaccinated birds. Phylogenetic analysis showed close relationships with global strains, highlighting the need for enhanced surveillance and control strategies.

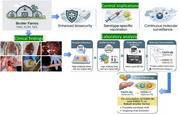

## 1 Introduction

Fowl adenoviruses (FAdVs) are major viral pathogens in poultry production and represent the genus *Aviadenovirus* within the family Adenoviridae. FAdVs are non‐enveloped, double‐stranded DNA viruses with a wide host range, and at least 12 recognized serotypes are known to induce clinical disease in chickens (Bayraktar et al. 2024; Harrach et al. 2011; Kaján et al. 2013; Kardoudi et al. 2025). FAdVs are the causative agents of several economically important diseases, notably inclusion body hepatitis (IBH), which has been identified in both broiler and breeder flocks worldwide (Niczyporuk et al. 2021; Schachner et al. 2018). IBH is characterized by hepatic necrosis with basophilic intranuclear inclusion bodies in hepatocytes, while adenoviral gizzard erosion (AGE) presents as erosive and ulcerative lesions of the gizzard, leading to impaired feed conversion, growth retardation, and increased mortality (Lim et al. 2011; Nakamura et al. 2011; Tamam et al. 2025; Vera‐Hernández et al. 2016). These diseases affect flock health and cause significant economic losses through reduced productivity, increased veterinary costs and trade impacts.

Globally, FAdV infections have been reported across Asia, Europe, Africa and the Americas, with distinct serotypes predominating in different regions. For instance, FAdV‐4 is mainly associated with hydropericardium‐hepatitis syndrome (HHS) in China and other parts of Asia; FAdV‐8b has emerged as a common serotype causing IBH outbreaks in Europe and parts of Asia, while FAdV‐11 is frequently reported in Africa (Mohamed Sohaimi and Ugwu 2021). This global serotype diversity underscores the importance of continuous surveillance and molecular characterization of circulating FAdVs to detect emerging variants and to inform vaccine strategies.

Over the past decade, there has been a notable increase in the number of FAdV‐associated clinical cases in commercial poultry in Malaysia. Retrospective analyses and outbreak investigations indicate that before 2012, confirmed FAdV infections were relatively rare and sporadic (Sabarudin et al. 2021). However, more recent studies from 2018 to 2023 document frequent detection and identification of diverse FAdV serotypes across multiple states in Malaysia (Mohamed Sohaimi and Ugwu 2021; Sabarudin et al. 2021). Studies conducted in Peninsular (West) Malaysia have identified FAdV‐8b (species E) as the predominant serotype responsible for severe IBH outbreaks in commercial broilers, with evidence of co‐circulating various FAdV serotypes across Malaysian chicken farms (Ahmed et al. 2025; Azlia et al. 2024; Bejo 2005; Mohamed Sohaimi and Bejo 2021). These findings are consistent with global trends, where FAdV‐8b has emerged as a common serotype responsible for widespread IBH cases in Asia and Europe. Given the documented predominance of FAdV‐8b in Peninsular Malaysia and the scarcity of molecular data from East Malaysia, hypothesized that FAdV serotype distribution in Sabah may differ from that reported in West Malaysia, potentially reflecting regional diversification or emerging serotype shifts. Moreover, the multiple FAdV serotypes may be co‐circulating within broiler farms and that serological antibody profiles would correlate with molecular detection rates, indicating active viral transmission dynamics.

Influencing factors, such as farm biosecurity practices, importation of day‐old chicks from external hatcheries, climatic conditions and management intensity, may all affect the dissemination and adaptation of FAdVs. Over the last decade, Sabah has experienced intensified poultry production, contributing significantly to broiler meat production in Malaysia. However, limited diagnostic facilities and insufficient surveillance activities in the state have hindered the timely detection and identification of new viral pathogens. Consequently, the molecular epidemiology of FAdV infection in Sabah remains poorly understood. Recent pathological cases and field surveillance in commercial broilers in Sabah indicate an increased number of IBH cases, with gross lesions characterized by pale, swollen livers, focal to diffuse hepatic necrosis and basophilic intranuclear inclusion bodies in hepatocytes (Mohamed Sohaimi et al. 2025; Stoute et al. 2025).

Therefore, this study was conducted to investigate the molecular and serological characteristics of FAdVs associated with IBH in broiler chickens from Sabah, Malaysia. Using PCR targeting the *hexon* gene, enzyme‐linked immunosorbent assay (ELISA)‐based antibody profiling, and sequence analyses, the research aimed to identify circulating FAdV serotypes, their prevalence, and assess potential shifts in serotype dynamics. Although FAdV‐8b has previously been reported as the dominant serotype in West Malaysia, information on circulating serotypes in East Malaysia, particularly Sabah, remains limited. To date, no comprehensive molecular or serological study has been conducted to confirm the identity of circulating FAdV serotypes and their correlation with previously reported Malaysian isolates.

## 2 Materials and Methods

### 2.1 Sample Collection

A total of 30 pooled tissue samples (liver and gizzard) and 60 blood samples were collected from commercial broiler chicken farms YAN, KON and NIS in Sabah, Malaysia, on the basis of the presence of clinical signs and gross pathological lesions consistent with IBH identified during routine veterinary diagnostic investigations. The selected farms were under routine diagnostic observation and were included as outbreak‐associated cases with suspected FAdV infection. All samples were aseptically collected by a licensed veterinarian during routine diagnostic farm visits. Sampling was conducted sequentially between August 2024 and February 2025, with each farm investigated at the time of clinical presentation. Accordingly, this study represents a cross‐sectional investigation of clinically affected flocks within a defined surveillance window rather than a population‐level prevalence survey or simultaneous multi‐farm sampling. Although this purposive outbreak‐based design may introduce selection bias and limit generalizability, it was methodologically appropriate for achieving the primary objective of molecular and serological characterization of FAdV in suspected IBH cases.

Blood samples were collected from the wing vein of broiler chickens from the same three farms, with 20 samples per farm at 35–40 days of harvesting age. Regarding vaccination history, the birds received NDV and IBV vaccines from the hatchery, administered at Day 1 (spray) and Day 10 (drinking water) using NDV clone and IBV Ma5 strain (Nobilis), respectively. After collection, blood samples were kept at room temperature to allow clotting and were then transported to the laboratory under chilled conditions. In the laboratory, the samples were centrifuged at 3000 rpm for 10 min, and the separated sera were stored at −20°C until analysis. Tissue samples were also transported to the laboratory under chilled conditions and stored at −80°C until processing. As the samples were collected as part of routine diagnostic, animal ethics approval was not required.

A priori power analysis was conducted assuming an expected FAdV prevalence of 30%–40%, with *α *= 0.05 and 80% power (*β *= 0.20). On the basis of these assumptions, a minimum of 9–10 pooled samples per farm was required to detect a ≥40% difference in prevalence among farms. Therefore, 10 pooled tissue samples per farm were included. Tissue pooling (liver and gizzard) was performed to enhance diagnostic efficiency and cost‐effectiveness while maintaining adequate sensitivity for flock‐level detection. As the primary objective was to determine farm‐level viral presence and serotype distribution rather than individual‐bird prevalence, pooled sampling was considered methodologically appropriate.

### 2.2 Serology Analysis

To assess flock immunity and exposure to FAdVs, a serological study was conducted using a commercial ELISA kit (Biocheck, the Netherlands). The ELISA was performed according to the manufacturer's instructions. Briefly, diluted serum samples, along with positive and negative controls, were added to antigen‐coated wells of a microtitre plate and incubated at room temperature. After washing to remove unbound antibodies, an enzyme‐conjugated anti‐chicken antibody was added, followed by substrate solution. The reaction was stopped using stop solution, and optical density (OD) values were measured at 405 nm using an ELISA reader. Results were expressed as sample‐to‐positive (S/P) ratios, antibody titres, geometric mean titres (GMT), and coefficient of variation (%CV). Flocks were categorized as negative, suspect or positive according to the Biocheck interpretation guidelines. Data were analysed to assess antibody distribution, flock immunity status and possible exposure to IBH.

### 2.3 DNA Extraction and PCR Detection

Genomic DNA was extracted from approximately 25 mg of each tissue sample using the QIAamp DNA Mini Kit (Qiagen, Germany) according to the manufacturer's protocol. DNA quality and concentration were measured using a NanoDrop spectrophotometer (Thermo Fisher Scientific, USA). FAdV detection was carried out by conventional PCR targeting a conserved region of the *hexon* gene, with the per stated primers used previously (Meulemans et al. 2001). Amplification was conducted in a 25 µL reaction mixture containing 1 × PCR buffer, 1.5 mM MgCl_2_, 200 µM dNTPs, 0.4 µM of each primer, 1 U Taq DNA polymerase (Thermo Fisher Scientific) and 100 ng template DNA. The primer pair Hexon A (forward: 5′‐CAARTTCAGRCAGACGGT‐3′) and Hexon B (reverse: 5′‐TAGTGATGMCGSGACATCAT‐3′) was used to amplify an approximately 897 bp fragment. PCR was performed under the following conditions: denaturation at 94°C for 2 min; 35 cycles of denaturation at 94°C for 30 s, annealing at 60°C for 1 min and extension at 72°C for 90 s; followed by a final extension at 72°C for 2 min. PCR products were visualized on a 1.5% agarose gel stained with ethidium bromide under UV illumination.

### 2.4 Molecular Detection and Sequencing

All PCR‐positive samples underwent partial sequencing of the *hexon* gene, which encodes the major capsid protein and is considered the gold standard for FAdV serotyping. PCR products were purified using the QIAquick PCR Purification Kit (Qiagen, Germany) and sequenced bidirectionally on an ABI 3730XL DNA Analyzer (Applied Biosystems, USA). The resulting chromatograms were assembled, edited and aligned using BioEdit v7.2 to generate consensus sequences for further phylogenetic analysis.

### 2.5 Sequence Alignment and Phylogenetic Analysis

To determine the evolutionary relationships among FAdV isolates, sequence alignment and phylogenetic analysis were performed based on the partial *hexon* gene sequences. Representative FAdV sequences from various countries were retrieved from GenBank and aligned with the sequences obtained in this study using MAFFT (v7.450) with the G‐INS‐i algorithm (BLOSUM62 scoring matrix; gap open penalty: 1.53; offset value: 0.123) implemented in Geneious Prime (v23.1.1, Biomatters Ltd., Auckland, New Zealand). A maximum likelihood (ML) phylogenetic tree was constructed in Geneious Prime with 1000 bootstrap replicates to assess the reliability of the inferred relationships. A human adenovirus 36 (HAdV‐36) sequence (GenBank accession no. GQ384080) was used as an out‐group to root the tree. The final tree was visualized and annotated using FigTree v1.4.4, with bootstrap support values presented as percentages along the nodes.

### 2.6 Statistical Analysis

Antibody titres for each farm were expressed as mean ± standard deviation (SD). Differences among farms were analysed using one‐way ANOVA, followed by Tukey's post hoc test, with *p *< 0.05 considered statistically significant. In the corresponding figures, error bars represent SD to show variation among individual birds.

## 3 Results

### 3.1 Gross Pathological Lesions

**FIGURE 1:**
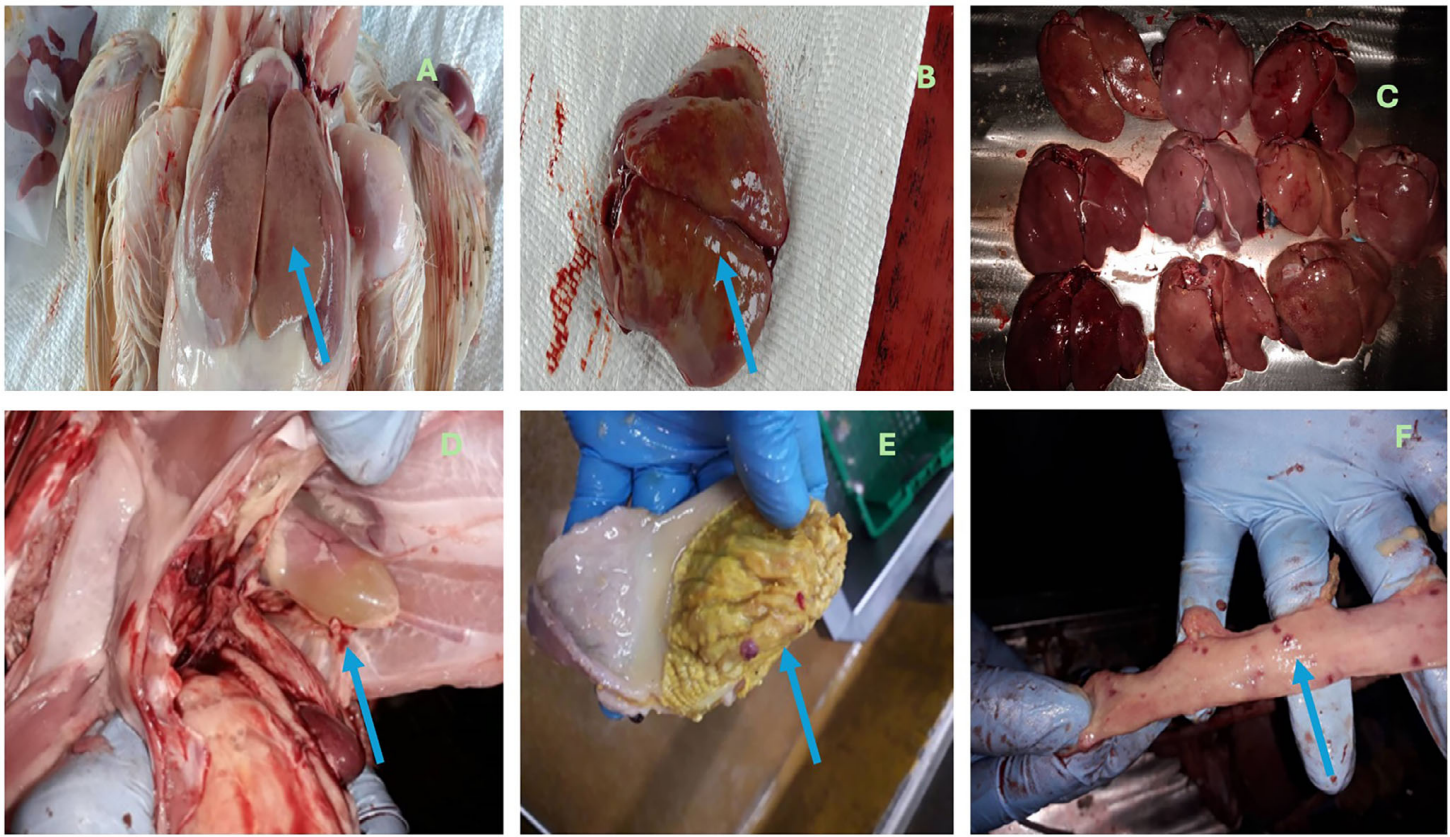
Gross pathological lesions observed in broiler chickens naturally infected with fowl adenovirus (FAdV): (A) enlarged, pale and friable liver with distinct reticular pattern; (B) multifocal hepatic necrosis with petechial haemorrhages on the liver surface; (C) comparison of multiple affected livers showing varying degrees of discolouration and enlargement; (D) the presence of hydropericardium; (E) erosive and ulcerative lesions in the gizzard lining consistent with AGE; and (F) petechial haemorrhagic in intestinal lining.

Broiler chickens naturally infected with FAdV exhibited characteristic gross lesions consistent with IBH and AGE. The study evaluated the overall occurrence of gross lesions across all birds, without recording the frequency of each specific lesion for every individual. Notably, all birds (100%) exhibited pale, friable livers with reticular patterns across the three farms (Figure 1A). Other lesions, including petechial with ecchymotic haemorrhages (Figure 1B), were observed but were not quantified on a per‐bird basis. Comparative assessment of multiple livers demonstrated varying degrees of discolouration and enlargement (Figure 1C). In several birds, straw‐coloured fluid accumulation was observed in the pericardial sac, indicative of hydropericardium (Figure 1D). Lesions in the gizzard consisted of erosive and ulcerative changes in the koilin layer, consistent with AGE (Figure 1E). Additionally, petechial haemorrhages in intestinal lining indicate supportive evidence of systemic viral infection, but the liver lesions are the hallmark for diagnosing IBH (Figure 1F).

### 3.2 Seroprevalence of Response to FAdV in Different Broiler Farms

**FIGURE 2:**
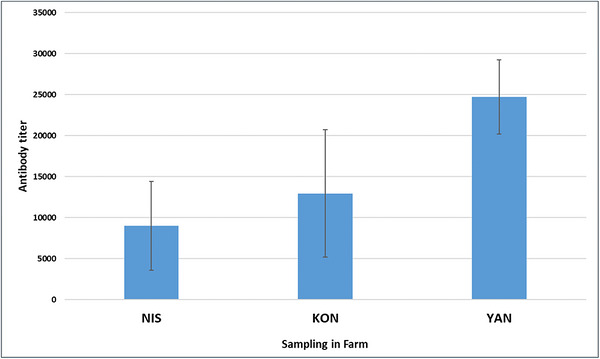
Comparison of mean antibody titres among broiler farms (NIS, KON and YAN). Data are presented as mean ± SD (*n *= 20 birds per farm). Differences among farms were analysed using one‐way ANOVA followed by Tukey's post hoc test.

The overall antibody response to FAdV varied considerably among the three farms, as illustrated in Figure S1. The distribution of antibody titres in the NIS, KON and YAN farms showed clear differences in immune response profiles, with the YAN farm exhibiting significantly higher and more uniform titre responses compared to the other two farm groups. Mean antibody titres differed significantly by location, with YAN Farm having the highest GMT of 24,716.5 ± 4516.1, followed by KON Farm (12,938.1 ± 7774.8) and NIS Farm (8993.9 ± 5437.9) (Figure 2).

### 3.3 Molecular Detection

PCR screening targeting the *hexon* gene of FAdV revealed variable detection rates among the all collected sample from three farms. Of the 30 pooled tissue samples (liver and gizzard) tested, 10 samples (33.3%, 95% CI: 17.3–52.8) were positive for FAdV, producing the expected 897 bp amplicon, which confirmed the molecular presence of FAdV in the flocks. The *hexon* gene, which encodes the major capsid protein, is widely recognized as the gold standard molecular marker for FAdV detection and serotyping. Among the three farm locations, YAN Farm showed the highest positivity rate, with 7 out of 10 samples testing positive (70%, 95% CI: 44.4–97.5), followed by KON Farm with 2 positive samples (20%, 95% CI: 2.5–55.6), and NIS Farm with 1 positive sample (10%, 95% CI: 0.3–44.5). The substantially higher detection rate observed in YAN suggests stronger viral circulation or more active FAdV infection within this flock compared to the other farms.

### 3.4 Sequence Analysis and Evolutionary Relationships of FAdVs

Partial sequences of the *hexon* gene of FAdV were successfully obtained from the analysed samples. After quality trimming of the raw sequencing reads, nucleotide sequences ranging from 564 to 810 bp were generated. These sequences represent partial fragments of the *hexon* gene, which is approximately 2.7–3.0 kb in length and encodes the major capsid protein responsible for important antigenic characteristics of the virus. The amplified region most likely encompasses portions of the L1 loop region of the *hexon* gene, which contains several hypervariable regions (HVRs) commonly used for molecular typing and phylogenetic characterization of FAdV strains. Alignment of the obtained sequences revealed nucleotide variability within this region, which is consistent with previously reported genetic variation in the L1 loop of the *hexon* gene. Such variability is expected, as the L1 loop contains the hypervariable segments HVR1–HVR4 that contribute to antigenic diversity among FAdV strains (Niczyporuk 2018).

**FIGURE 3:**
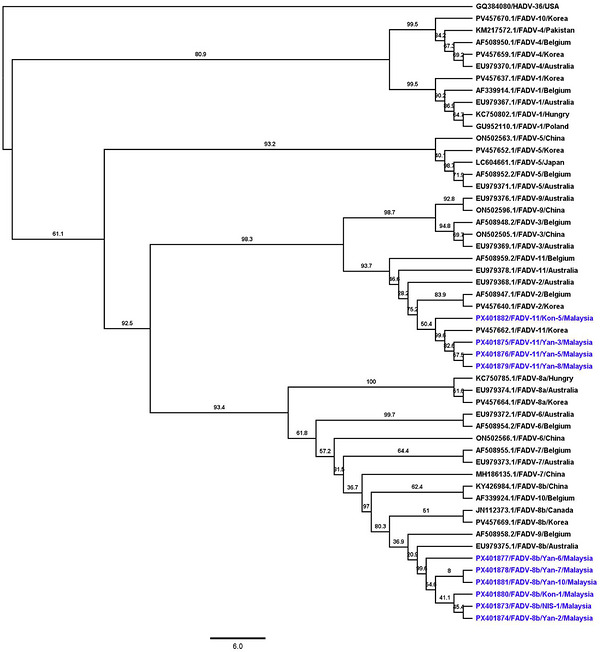
A phylogenetic tree illustrating the evolutionary relationships among selected FADV isolates was constructed. Partial *hexon* gene sequences from representative FADV strains originating from various countries were aligned using MAFFT (v7.450) with the G‐INS‐i algorithm (BLOSUM62 scoring matrix; gap open penalty: 1.53; offset value: 0.123) implemented in Geneious Prime (v2025.1.1, Biomatters Ltd., Auckland, New Zealand). A maximum likelihood tree was generated in Geneious Prime using the GTR substitution model, with 1000 bootstrap replicates. A human adenovirus 36 (HAdV‐36) sequence (GenBank accession no. GQ384080) used as the out‐group. Branch tip labels denote the GenBank accession number, virus name, and country of origin. Sequences obtained in this study are highlighted in blue. The tree was visualized using FigTree v1.4.4, and bootstrap values are shown as percentages at the nodes.

Sequence alignment of partial *hexon* gene sequences was performed in Geneious Prime (version 2025.1.1) using MAFFT (version 7.450) with the G‐INS‐i algorithm, applying a gap opening penalty of 1.53 and an offset value of 0.123. The alignment revealed sequence homology ranging from 60.51% to 99.78%, indicating substantial diversity among FAdV circulating in Malaysia (Figure S2). Additionally, alignment of selected FAdV sequences from various geographical regions with our isolates demonstrated further divergence, with homology ranging from 43.71% to 99.83% (Figure S3). Phylogenetic analysis based on partial *hexon* gene sequences amplified from PCR‐positive samples was performed alongside representative reference sequences from GenBank to infer the evolutionary relationships of the Malaysian FAdV isolates (Figure 3). The Sabah isolates (GenBank accession numbers PX401882, PX401875, PX401876, PX401877, PX401878 and PX401883) segregated into two major clades corresponding to FAdV species D and E, consistent with serotypes FAdV‐11 and FAdV‐8b, respectively. Isolates PX401882/FAdV‐11/Kon‐5/Malaysia, PX401875/FAdV‐11/Yan‐3/Malaysia and PX401876/FAdV‐11/Yan‐8/Malaysia formed a robust monophyletic cluster with reference FAdV‐11 strains from Korea (AF508949) and Belgium (AF508950), supported by strong bootstrap values (94%–99%). Conversely, isolates PX401877/FAdV‐8b/Yan‐7/Malaysia, PX401878/FAdV‐8b/Yan‐10/Malaysia, and PX401883/FAdV‐8b/Nis‐1/Malaysia grouped tightly with Australian (KC705875) and Chinese (MH188135) FAdV‐8b strains, with high nodal support (>97%). Despite close phylogenetic affinities with these geographically diverse reference strains, minor sequence divergence observed among Sabah isolates within each serotype suggests ongoing intra‐serotype evolution, potentially driven by local adaptation or host immune selection pressures. These findings imply that FAdV populations circulating in Malaysian poultry may represent regionally evolving lineages that maintain global phylogenetic connectivity while exhibiting localized diversification. In summary, both FAdV‐11 and FAdV‐8b were detected across the sampled farms, with farm‐specific positivity as follows: Farm KON: FAdV‐8b 1/10 (10%), FAdV‐11 1/10 (10%); Farm YAN: FAdV‐8b 3/10 (30%), FAdV‐11 4/10 (40%); and Farm NIS: FAdV‐8b 0/10 (0%), FAdV‐11 1/10 (10%). Phylogenetic analysis (Figure 3) confirmed these identifications, indicating co‐circulation of both serotypes rather than strict farm‐specific associations, which is epidemiologically relevant.

## 4 Discussion

Clinically, the affected flocks exhibited increased mortality, depression, ruffled feathers and poor feed conversion efficiency. Post‐mortem examination revealed enlarged, friable and pale livers with focal to diffuse necrosis and characteristic basophilic intranuclear inclusion bodies in hepatocytes, consistent with typical IBH lesions. Gizzard erosions were also observed in some birds, suggesting FAdV involvement in multiple organ systems. These pathological changes correspond with those previously described in broiler infections caused by FAdV (Niczyporuk et al. 2020; Vera‐Hernández et al. 2016). In our study, gizzard erosions were observed in multiple broiler chickens; however, PCR and sequence analysis did not detect FAdV‐1 (species A), which is classically associated with AGE. The detected serotypes, FAdV‐D (FAdV‐11) and FAdV‐E (FAdV‐8b), are predominantly linked to IBH, some strains within those species have been associated with gizzard erosion (Mirzazadeh et al. 2021). Due to co‐circulation of these serotypes in the affected flocks and the outbreak‐based sampling design, it was not possible to definitively determine which serotype was responsible for the observed gizzard lesions. To address differential diagnosis, other potential causes of gizzard lesions, including bacterial or fungal toxin, nutritional or management‐related causes, and other viral agents, were considered during routine diagnostic investigations. No evidence of other pathogens was detected, suggesting that the gizzard lesions were likely associated with FAdV infection, although the specific causal serotype could not be confirmed.

The concurrent detection of both FAdV‐8b and FAdV‐11 within the same production cycle suggests either co‐circulation or sequential infection, both of which may exacerbate disease severity. Although co‐infections with immunosuppressive agents were not assessed, such interactions could have contributed to the clinical outcomes. Phylogenetic and molecular analyses identified two predominant serotypes‐FAdV‐8b (species E) and FAdV‐11 (species D). Previous study reported that FAdV‐8b was the predominant and sole serotype detected in broiler and breeder flocks in West Malaysia, particularly in Selangor (Sabarudin et al. 2021). Similarly, earlier outbreaks in West Malaysia were largely attributed to FAdV‐8b (Bejo 2005; Lim et al. 2011). In contrast, our study demonstrates the co‐circulation of both FAdV‐8b and FAdV‐11 in Sabah, East Malaysia, indicating recent diversification and regional variation in FAdV ecology.

The *hexon* gene is the largest and one of the most conserved structural genes of FAdV, with a length of approximately 2.7–3.0 kb, and it encodes the major capsid protein responsible for important antigenic and immunogenic properties of the virus. In the present study, after quality trimming of the sequencing reads, nucleotide sequences of varying lengths (564–810 bp) were obtained. These sequences represent only partial fragments of the *hexon* gene and most likely encompass portions of the L1 loop region, which contains several HVRs widely used for molecular typing and phylogenetic characterization of FAdV strains. Because the obtained sequences cover only a limited portion of the *hexon* gene, reconstruction of the complete open reading frame (ORF) was not possible from our dataset. In addition, the variable length of the nucleotide fragments restricts comprehensive protein‐level analyses of the hexon protein, including full amino acid sequence comparison or structural interpretation. Consequently, the present analysis was primarily limited to nucleotide‐based comparison and phylogenetic inference using the available partial sequences. Despite these limitations, the L1 loop region remains highly informative for strain differentiation, as it contains the HVR1–HVR4 segments that contribute significantly to antigenic variability and serotype identification. Previous studies have demonstrated that sequence variation within these HVRs plays a critical role in distinguishing field strains and understanding their molecular diversity. In particular, Niczyporuk (2018) provided a detailed characterization of the L1 HVR1–HVR4 regions of adenovirus field strains isolated in Poland, highlighting the importance of this region for molecular epidemiological investigations. Therefore, although the sequences obtained in this study represent only partial fragments of the *hexon* gene, they still provide valuable information for the molecular characterization and comparative analysis of circulating FAdV strains.

Phylogenetic analysis further revealed that the Sabah isolates clustered into two major clades corresponding to FAdV species D (FAdV‐11) and species E (FAdV‐8b), consistent with molecular typing results. Strong bootstrap support (94%–99% for FAdV‐11 and >97% for FAdV‐8b) and high nucleotide identity (98%–99%) with reference strains from Korea, Belgium, China and Australia indicate that these Malaysian isolates are closely related to globally circulating lineages. Notably, the FAdV‐11 isolates formed a distinct monophyletic cluster separate from the historically dominant FAdV‐8b lineage, suggesting a possible serotype shift within Sabah's poultry population. This phylogenetic analysis suggests the possibility of emerging genetic variants; however, further serological testing, including SNT, is required to confirm any serotype‐specific shift. This emerging pattern mirrors global trends reported in China, India and Europe, where FAdV‐11 has replaced or co‐circulated with FAdV‐8b as a major cause of IBH (Kaján et al. 2013; Mirzazadeh et al. 2021; Sadekuzzaman et al. 2024; Vera‐Hernández et al. 2016; Wajid et al. 2018).

Minor sequence divergence observed among isolates within each serotype suggests ongoing intra‐serotype evolution, likely driven by host immune pressure, viral adaptation or environmental selection within intensive production systems. The co‐existence of both FAdV serotypes in Sabah is epidemiologically important, as it raises the potential for recombination and incomplete cross‐protection, which could facilitate the emergence of novel variants with altered virulence or transmission dynamics.

The serology results indicate that YAN Farm had a stronger and more uniform antibody response, suggesting a higher level of FAdV exposure or active immune stimulation, likely due to natural infection or recent vaccination. In contrast, the lower and more variable titres observed at KON and NIS Farms may reflect differences in virus circulation or flock‐level immune responses. Although descriptive trends showed higher antibody titres at YAN compared to the other farms, statistical analysis found no significant differences (*p* > 0.05) among the three farms. This suggests that, within the sampled populations, the observed variations in serological titres may not be driven solely by farm‐level factors but could instead be influenced by individual bird‐level variation or management‐related variables.

A strong correlation between molecular detection and serological responses was evident across the three farms. At YAN Farm, the highest PCR positivity (70%) corresponded with the highest mean antibody titre (24,716.5 ± 4516.1), reflecting active viral circulation and strong immune stimulation. In contrast, KON Farm showed moderate PCR positivity (20%) and intermediate antibody levels (12,938.1 ± 7774.8), suggesting a resolving infection or recent exposure, whereas NIS Farm exhibited the lowest PCR positivity (10%) and antibody titre (8993.9 ± 5437.9), indicating limited viral activity. These results highlight that combined molecular and serological surveillance offers a more comprehensive understanding of FAdV infection dynamics within poultry populations.

In our study, across all sampled farms, FAdV‐8b was detected in 4/10 samples (40%) and FAdV‐11 in 6/10 samples (60%). In contrast, previous studies from West Malaysia reported FAdV‐8b as the predominant serotype, often accounting for most isolates with limited detection of other serotypes. This comparison highlights that, while FAdV‐8b remains prevalent, our findings reveal a substantial presence of FAdV‐11 and co‐circulation of both serotypes, which was not documented in earlier reports.

Serological screening using ELISA revealed widespread exposure to FAdV, indicating that subclinical infections are common among broilers. The high antibody titres detected in apparently healthy birds suggest early exposure in the production cycle, likely resulting from vertical transmission from infected breeder flocks or rapid horizontal transmission within farms. Vertical transmission from infected breeders to progeny is well documented and remains a major route of infection leading to early onset IBH in young broilers (Niczyporuk et al. 2020; Schachner et al. 2018). These findings are consistent with previous observations in West Malaysia, where 60%–80% seropositivity was reported in commercial poultry populations (Bejo 2005). The high seroprevalence observed in Sabah suggests that FAdV infection may be endemic in the region, maintained by incomplete biosecurity, inadequate litter management, and the inter‐regional movement of chicks and hatching eggs. These findings highlight the urgent need for strengthened biosecurity measures, regular molecular surveillance and the implementation of serotype‐specific vaccination strategies to control FAdV transmission and evolution in Malaysian poultry. Although biosecurity was not systematically evaluated in this study, inadequate farm biosecurity is widely recognized as a potential risk factor for viral transmission in poultry production systems.

Although our study focused exclusively on IBH, occasional field reports describe birds presenting both IBH and AGE lesions. Although AGE was not systematically investigated in this work, literature suggests that such co‐manifestation may result from co‐infection with multiple FAdV serotypes or from secondary stress factors, including management and nutritional challenges. Our findings of co‐circulation of FAdV‐8b and FAdV‐11 across farms highlight the potential for multiple serotypes to contribute to disease complexity, but further studies are needed to elucidate the relationship between IBH and AGE lesions in poultry. On the other hand, concurrent detection of FAdV‐8b and FAdV‐11 in organ and blood samples collected at the same time indicates that both serotypes were present in the flocks. However, because sampling was performed at a single time point and no longitudinal or seroconversion monitoring was conducted, it is not possible to determine whether the two serotypes were circulating simultaneously (co‐circulation) or if one infection preceded the other (sequential infection). The current study did not include experimental assessment of cross‑protection between FAdV‑8b and FAdV‑11. Future longitudinal studies, actual cross‐protective responses and recombination potential are needed to clarify the dynamics and sequence of FAdV infections within flocks.

Our findings have important implications for vaccination strategies. Currently, commercial FAdV vaccines in Malaysia primarily target FAdV‐8b. The co‐circulation of FAdV‐11 in Sabah suggests that monovalent vaccines may provide incomplete protection, as cross‐protection between serotypes is not guaranteed. Therefore, vaccination programmes should consider serotype‐specific or multivalent formulations to reduce IBH incidence and limit viral circulation. Future studies assessing actual cross‐protection through challenge trials would be useful to confirm the protective efficacy of existing vaccines against multiple circulating serotypes. Overall, our study demonstrates co‐circulation of FAdV‐8b and FAdV‐11 in Sabah broilers, underlining the need for serotype‐specific vaccination strategies, strengthened biosecurity, and continuous molecular and serological surveillance to effectively control IBH in Malaysian poultry farms.

Given the demonstrated co‐circulation of FAdV‐8b and FAdV‐11 in Sabah, and to address the unresolved question of cross‐protection, future in vivo challenge studies are warranted using well‐characterized isolates of both serotypes. Such studies should employ controlled experimental infection models in specific pathogen‐free (SPF) or commercial broilers, incorporating both homologous and heterologous challenge groups to evaluate clinical outcomes, viral load, tissue tropism and serological responses. This approach would enable direct assessment of the extent and durability of cross‐protective immunity between these serotypes and inform the design of effective monovalent versus multivalent vaccination strategies under field‐relevant conditions.

## 5 Conclusions

This study documents the concurrent circulation of FAdV‐8b (species E) and FAdV‐11 (species D) in broiler flocks in Sabah, Malaysia, thereby providing new regional epidemiological data on FAdVs. Phylogenetic and serological analyses revealed active viral transmission, high exposure rates and close evolutionary relationships with globally circulating lineages. Given the higher detection rate of FAdV‐11 (60%) compared to FAdV‐8b (40%), vaccine strategies in East Malaysian region should be prioritized to mitigate circulating FAdV‐11, while considering multivalent formulations due to the concurrent circulation of multiple serotypes. In addition to molecular detection, integrating routine serological monitoring would provide complementary insights into flock‐level exposure, immune status and potential seroconversion patterns. Furthermore, systematic surveillance is ideally conducted once per production cycle or during suspected outbreaks that would enable early detection of shifting serotype dynamics and support timely, evidence‐based control strategies.

## Author Contributions

Conceptualization: Md. Safiul Alam Bhuiyan and Subir Sarker. Methodology: Md. Safiul Alam Bhuiyan. Software: Md. Safiul Alam Bhuiyan and Babu Kanti Nath. Validation: Md. Safiul Alam Bhuiyan and Subir Sarker. Formal analysis: Md. Safiul Alam Bhuiyan, Suman Das Gupta, and Babu Kanti Nath. Investigation: Md. Safiul Alam Bhuiyan. Resources: Md. Safiul Alam Bhuiyan and Subir Sarker. Data curation: Md. Safiul Alam Bhuiyan and Babu Kanti Nath. Writing – original draft preparation: Md. Safiul Alam Bhuiyan. Writing – review and editing: Md. Safiul Alam Bhuiyan, Babu Kanti Nath, Lum Mok Sam, Krishnan Nair Balakrishnan, Suman Das Gupta and Subir Sarker. Visualization: Md. Safiul Alam Bhuiyan and Babu Kanti Nath. Supervision: Subir Sarker. Project administration: Md. Safiul Alam Bhuiyan and Subir Sarker. All authors have read and agreed to the published version of the manuscript.

## Funding

Subir Sarker is the recipient of an Australian Research Council Discovery Early Career Researcher Award (Grant DE200100367) funded by Australian Government.

## Ethics Statement

The authors have nothing to report.

## Consent

The authors have nothing to report.

## Conflicts of Interest

The authors declare no conflicts of interest.

## Supporting information



Supporting Information: The following supporting information can be downloaded at https://www.mdpi.com/article/doi/s1, **Supporting Figure 1**: Distribution of antibody titre groups for FAdV across three commercial broiler breeder farms. **Supporting Figure 2**: Sequence alignment of partial *hexon* gene sequences from the ten newly sequenced FAdV isolates identified in this study was performed in Geneious Prime (version 2025.1.1) using MAFFT (version 7.450) with the G‐INS‐i algorithm, applying a gap opening penalty of 1.53 and an offset value of 0.123. The alignment revealed sequence homology ranging from 60.51% to 99.78%. **Supporting Figure 3**: Nucleotide sequence alignment of partial *hexon* gene sequences from 51 selected FAdV sequences, including the 10 newly identified isolates from this study, was performed in Geneious Prime (version 2025.1.1) using MAFFT (version 7.450) with the G‐INS‐i algorithm, applying a gap opening penalty of 1.53 and an offset value of 0.123. The alignment revealed sequence homology ranging from 43.71% to 99.83%.

## Data Availability

The data that support the findings of this study are openly available in NCBI GenBank at https://www.ncbi.nlm.nih.gov/genbank/, reference number PX401873‐PX401882.
