# Supplementary material for: Integrating Serological and Molecular Data to Characterize Fowl Adenovirus Associated With Inclusion Body Hepatitis in Broiler Chickens From Malaysia
Source: Vet Med Sci. 2026 Jun 23;12(4):e71033. doi: 10.1002/vms3.71033 (PMC13288169; doi:10.1002/vms3.71033)
Supplement: Supplementary file 1 — Supporting Information: The following supporting information can be downloaded at https://www.mdpi.com/article/doi/s1, Supporting Figure 1: Distribution of antibody titre groups for FAdV across three commercial broiler breeder farms. Supporting Figure 2: Sequence alignment of partial hexon gene sequences from the ten newly sequenced FAdV isolates identified in this study was performed in Geneious Prime (version 2025.1.1) using MAFFT (version 7.450) with the G‐INS‐i algorithm, applying a gap opening penalty of 1.53 and an offset value of 0.123. The alignment revealed sequence homology ranging from 60.51% to 99.78%. Supporting Figure 3: Nucleotide sequence alignment of partial hexon gene sequences from 51 selected FAdV sequences, including the 10 newly identified isolates from this study, was performed in Geneious Prime (version 2025.1.1) using MAFFT (version 7.450) with the G‐INS‐i algorithm, applying a gap opening penalty of 1.53 and an offset value of 0.123. The alignment revealed sequence homology ranging from 43.71% to 99.83%. [file VMS3-12-e71033-s001.pdf]

*Supplementary File*

# Integrating Serological and Molecular Data to Characterize Fowl Adenovirus Associated with Inclusion Body Hepatitis in Broiler Chickens from Malaysia

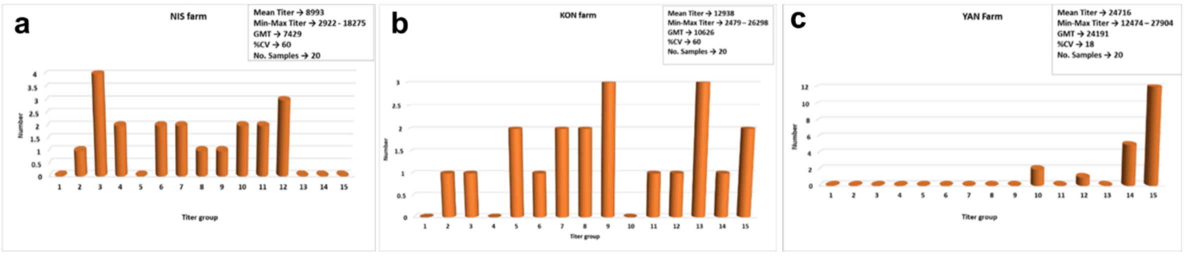


**Supplementary Figure S1**: Distribution of antibody titer groups for FAdV across three commercial broiler breeder farms: (a) NIS farm, (b) KON farm, and (c) YAN farm. Each bar represents the number of samples in each titer group. For each farm, basic statistics were calculated, including mean titer, minimum and maximum titers, geometric mean titer (GMT), coefficient of variation (%CV), and sample size. The figure shows considerable variation among farms in the magnitude of immune response, with higher and more consistent titers observed in YAN farm compared to NIS and KON farms.

| | PX401873/ | PX401880/ | PX401874/ | PX401878/ | PX401877/ | PX401881/ | PX401875/ | PX401876/ | PX401879/ | PX401882/ |
|---|---|---|---|---|---|---|---|---|---|---|
| PX401873/FADV-8b/NIS-1/Malaysia | | 99.59 | 99.72 | 99.17 | 98.39 | 95.5 | 70.64 | 70.45 | 70.45 | 64.54 |
| PX401880/FADV-8b/Kon-1/Malaysia | 99.59 | | 99.57 | 92.51 | 91.6 | 93.84 | 66.29 | 66.74 | 66.75 | 61.37 |
| PX401874/FADV-8b/Yan-2/Malaysia | 99.72 | 99.57 | | 99.57 | 98.98 | 99.03 | 69.93 | 69.8 | 69.71 | 65.64 |
| PX401878/FADV-8b/Yan-7/Malaysia | 99.17 | 92.51 | 99.57 | | 98.92 | 94.54 | 71.18 | 70.78 | 70.82 | 60.7 |
| PX401877/FADV-8b/Yan-6/Malaysia | 98.39 | 91.6 | 98.98 | 98.92 | | 94.22 | 70.31 | 70.2 | 70.24 | 60.51 |
| PX401881/FADV-8b/Yan-10/Malaysia | 95.5 | 93.84 | 99.03 | 94.54 | 94.22 | | 64.63 | 64.4 | 64.42 | 62.52 |
| PX401875/FADV-11/Yan-3/Malaysia | 70.64 | 66.29 | 69.93 | 71.18 | 70.31 | 64.63 | | 99.78 | 99.75 | 88.47 |
| PX401876/FADV-11/Yan-5/Malaysia | 70.45 | 66.74 | 69.8 | 70.78 | 70.2 | 64.4 | 99.78 | | 99.52 | 88.21 |
| PX401879/FADV-11/Yan-8/Malaysia | 70.45 | 66.75 | 69.71 | 70.82 | 70.24 | 64.42 | 99.75 | 99.52 | | 88.19 |
| PX401882/FADV-11/Kon-5/Malaysia | 64.54 | 61.37 | 65.64 | 60.7 | 60.51 | 62.52 | 88.47 | 88.21 | 88.19 | |

**Supplementary Figure S2**: Sequence alignment of partial hexon gene sequences from the ten newly sequenced FAdV isolates identified in this study was performed in Geneious Prime (version 2025.1.1) using MAFFT (version 7.450) with the G-INS-i algorithm, applying a gap opening penalty of 1.53 and an offset value of 0.123. The alignment revealed sequence homology ranging from 60.51% to 99.78%.

| | AF339914. | AF339924. | AF508947. | AF508948. | AF508950. | AF508952. | AF508954. | AF508955. | AF508958. | AF508959. | EU979367. | EU979368. | EU979369. | EU979370. | EU979371. | EU979372. | EU979373. | EU979374. | EU979375. | EU979376. | EU979378. | GU952110 | JN112373. | KC750785. | KC750802. | KM217572 | KY426984. | LC604661. | MH186135 | ON502505 | ON502563 | ON502566 | ON502596 | PV457637. | PV457640. | PV457652. | PV457659. | PV457662. | PV457664. | PV457669. | PV457670. | PX401873/ | PX401874/ | PX401875/ | PX401876/ | PX401877/ | PX401878/ | PX401879/ | PX401880/ | PX401881/ | PX401882/ |
|---|---|---|---|---|---|---|---|---|---|---|---|---|---|---|---|---|---|---|---|---|---|---|---|---|---|---|---|---|---|---|---|---|---|---|---|---|---|---|---|---|---|---|---|---|---|---|---|---|---|---|---|
| AF339914.1/FADV-1/Belgium | | 67.21 | 65.31 | 66.47 | 67.76 | 69.02 | 68.14 | 69.53 | 67.56 | 66.01 | 99.83 | 59.73 | 61.24 | 63.24 | 63.12 | 62.92 | 63.26 | 61.41 | 61.07 | 61.24 | 60.07 | 99.46 | 66.75 | 59.2 | 99.63 | 61.4 | 57.67 | 60.67 | 59.73 | 60.86 | 61.56 | 64.02 | 64.49 | 94.37 | 61 | 64.62 | 63.79 | 61.58 | 64.14 | 63.91 | 64.37 | 64 | 63.67 | 60.09 | 63.53 | 62.4 | 62.31 | 63.5 | 56.84 | 48.71 | 46.29 |
| AF339924.1/FADV-10/Belgium | 67.21 | | 72.91 | 71.74 | 63.15 | 70.79 | 85.35 | 91.05 | 97.67 | 72.56 | 60.91 | 68.12 | 67.45 | 56.93 | 64.29 | 81.54 | 88.42 | 82.21 | 97.48 | 67.79 | 67.95 | 65.18 | 97.9 | 80.33 | 58.36 | 57.09 | 92.34 | 62.34 | 82.18 | 67.27 | 63.36 | 83.51 | 70.76 | 63.56 | 68.72 | 66.97 | 59.79 | 68.26 | 82.09 | 93.49 | 59.44 | 96.14 | 96.49 | 66.39 | 70.5 | 90.9 | 90.82 | 70.47 | 84.57 | 77.89 | 52.78 |
| AF508947.1/FADV-2/Belgium | 65.31 | 72.91 | | 77.45 | 63.91 | 69.94 | 73.95 | 73.02 | 72.21 | 95.09 | 59.4 | 93.93 | 72.34 | 57.21 | 65.61 | 70.81 | 69.3 | 69.46 | 68.29 | 73.86 | 94.1 | 62.48 | 71.57 | 67.58 | 57.09 | 57.26 | 64.75 | 61.99 | 64.6 | 71.9 | 65.64 | 70.31 | 76.68 | 61.66 | 91.47 | 67.05 | 59.95 | 91.24 | 69.73 | 68.68 | 59.6 | 70.62 | 70.45 | 89.57 | 95.2 | 67.2 | 67.02 | 95.17 | 62.5 | 55.14 | 75.04 |
| AF508948.2/FADV-3/Belgium | 66.47 | 71.74 | 77.45 | | 62.4 | 68.67 | 73.02 | 72.09 | 72.09 | 79.79 | 61.58 | 74.87 | 99.83 | 56.21 | 64.12 | 69.8 | 67.95 | 66.78 | 68.12 | 94.1 | 75.38 | 64.24 | 71.45 | 65.03 | 58.91 | 56.05 | 64.31 | 60.38 | 63.86 | 98.68 | 63.68 | 70.01 | 94.5 | 62.82 | 73.6 | 66.24 | 58.67 | 74.18 | 67.75 | 68.34 | 59.25 | 70.07 | 70.3 | 72.75 | 77.13 | 66.85 | 66.55 | 77.1 | 61.91 | 54.71 | 58.82 |
| AF508950.1/FADV-4/Belgium | 67.76 | 63.15 | 63.91 | 62.4 | | 65.66 | 64.31 | 65.01 | 63.5 | 63.1 | 63.07 | 56.38 | 56.04 | 98.79 | 59.47 | 57.93 | 58.6 | 58.6 | 56.59 | 56.21 | 56.21 | 65.04 | 61.7 | 56.7 | 60.88 | 92.29 | 54.77 | 58.04 | 56.68 | 56.91 | 58.96 | 59.7 | 60.88 | 63.63 | 60.07 | 60.92 | 93.58 | 59.37 | 60.9 | 59.4 | 92.75 | 59.07 | 58.17 | 57.97 | 61.63 | 58.21 | 57.86 | 61.47 | 52.76 | 45.8 | 43.71 |
| AF508952.2/FADV-5/Belgium | 69.02 | 70.79 | 69.94 | 68.67 | 65.66 | | 72.98 | 72.29 | 70.9 | 69.6 | 63.29 | 64.12 | 63.95 | 59.63 | 99.83 | 67.44 | 66.94 | 66.61 | 64.29 | 63.62 | 64.29 | 66.13 | 69.69 | 64.86 | 61.51 | 59.2 | 62.28 | 93.71 | 63.45 | 63.36 | 85.83 | 68.05 | 66.75 | 64.93 | 65.78 | 89.13 | 61.5 | 65.55 | 68.67 | 67.05 | 61.73 | 67.31 | 66.76 | 63.77 | 67.7 | 65.45 | 65.15 | 67.67 | 59.95 | 52.41 | 50.57 |
| AF508954.2/FADV-6/Belgium | 68.14 | 85.35 | 73.95 | 73.02 | 64.31 | 72.98 | | 88.02 | 84.77 | 74.3 | 62.92 | 71.31 | 69.46 | 58.43 | 67.44 | 99.83 | 85.74 | 82.38 | 81.04 | 69.3 | 70.97 | 66.26 | 84.05 | 81.42 | 60.73 | 58.56 | 75.99 | 65.11 | 78.94 | 69.24 | 67.43 | 89.36 | 71.83 | 64.38 | 70.58 | 68.71 | 60.83 | 70.12 | 81.74 | 80.35 | 60.6 | 82.9 | 82.94 | 68.4 | 72.78 | 78.48 | 78.35 | 72.75 | 73.03 | 64.62 | 55.21 |
| AF508955.1/FADV-7/Belgium | 69.53 | 91.05 | 73.02 | 72.09 | 65.01 | 72.29 | 88.02 | | 90.81 | 73.02 | 63.42 | 69.63 | 67.79 | 59.1 | 66.78 | 85.91 | 100 | 82.89 | 88.26 | 68.29 | 68.96 | 67.34 | 90.23 | 81.06 | 61.09 | 59.22 | 83.21 | 64.23 | 91.31 | 67.76 | 66.94 | 91 | 71.16 | 66.01 | 69.3 | 68.36 | 61.53 | 68.6 | 82.91 | 86.28 | 60.72 | 88.83 | 88.17 | 66.75 | 70.88 | 84.34 | 84.24 | 70.85 | 78.33 | 71.04 | 53.35 |
| AF508958.2/FADV-9/Belgium | 67.56 | 97.67 | 72.21 | 72.09 | 63.5 | 70.9 | 84.77 | 90.81 | | 72.21 | 61.24 | 68.12 | 67.95 | 56.93 | 63.95 | 81.04 | 88.09 | 81.04 | 99.83 | 68.12 | 67.95 | 65.45 | 99.38 | 79.05 | 58.73 | 57.22 | 91.61 | 62.34 | 82.18 | 67.76 | 64.5 | 82.91 | 71.03 | 63.91 | 68.6 | 66.86 | 59.79 | 67.91 | 81.28 | 94.65 | 59.56 | 97.66 | 97.88 | 66.04 | 70.12 | 92.3 | 92.24 | 70.09 | 85.87 | 79.6 | 52.78 |
| AF508959.2/FADV-11/Belgium | 66.01 | 72.56 | 95.09 | 79.79 | 63.1 | 69.6 | 74.3 | 73.02 | 72.21 | | 60.4 | 98.82 | 75.38 | 56.21 | 64.29 | 70.97 | 68.96 | 69.8 | 67.95 | 76.39 | 99.83 | 63.29 | 71.57 | 67.94 | 58 | 56.72 | 63.86 | 61.11 | 64.31 | 74.88 | 65.31 | 70.76 | 79.22 | 62.24 | 91.94 | 66.71 | 59.25 | 92.87 | 70.2 | 68.68 | 59.14 | 70.48 | 70.3 | 91.23 | 96.98 | 66.97 | 66.9 | 96.95 | 62.5 | 54.43 | 79.05 |
| EU979367.1/FADV-1/Australia | 99.83 | 60.91 | 59.4 | 61.58 | 63.07 | 63.29 | 62.92 | 63.42 | 61.24 | 60.4 | | 59.9 | 61.41 | 63.41 | 63.29 | 63.09 | 63.42 | 61.58 | 61.24 | 61.41 | 60.23 | 99.49 | 61.07 | 59.38 | 99.82 | 62.9 | 57.36 | 61.19 | 60.57 | 59.43 | 59.14 | 61.49 | 61.58 | 98.81 | 59.4 | 62.62 | 63.74 | 59.73 | 61.58 | 61.24 | 64.25 | 60.97 | 60.16 | 59.73 | 59.51 | 60.73 | 60.93 | 59.53 | 61.24 | 58.88 | 57.97 |
| EU979368.1/FADV-2/Australia | 59.73 | 68.12 | 93.93 | 74.87 | 56.38 | 64.12 | 71.31 | 69.63 | 68.12 | 98.82 | 59.9 | | 74.7 | 56.54 | 64.29 | 71.48 | 69.63 | 69.97 | 68.12 | 76.05 | 98.99 | 59.73 | 68.29 | 68.12 | 57.64 | 56.71 | 66.6 | 61.57 | 66.98 | 72.87 | 63.81 | 69.26 | 76.05 | 60.07 | 95.95 | 65.45 | 56.88 | 96.96 | 69.97 | 68.46 | 56.71 | 68.17 | 67.28 | 96.96 | 96.68 | 67.72 | 68.11 | 96.64 | 68.46 | 66.12 | 98.18 |
| EU979369.1/FADV-3/Australia | 61.24 | 67.45 | 72.34 | 99.83 | 56.04 | 63.95 | 69.46 | 67.79 | 67.95 | 75.38 | 61.41 | 74.7 | | 56.38 | 63.95 | 69.63 | 67.79 | 66.61 | 67.95 | 94.27 | 75.21 | 61.24 | 67.79 | 65.03 | 58.91 | 56.54 | 66.42 | 61.57 | 65.47 | 98.67 | 61.75 | 67.91 | 94.1 | 61.41 | 73.19 | 65.61 | 56.38 | 73.69 | 66.61 | 67.95 | 56.88 | 67.5 | 67.09 | 73.69 | 73.44 | 67.22 | 67.45 | 73.45 | 67.79 | 65.94 | 73.04 |
| EU979370.1/FADV-4/Australia | 63.24 | 56.93 | 57.21 | 56.21 | 98.79 | 59.63 | 58.43 | 59.1 | 56.93 | 56.21 | 63.41 | 56.54 | 56.38 | | 59.8 | 58.6 | 59.1 | 58.76 | 57.1 | 56.54 | 56.38 | 62.9 | 56.93 | 56.88 | 61.24 | 97.06 | 53.85 | 57.46 | 56.47 | 54.72 | 56.34 | 58.15 | 56.71 | 63.41 | 57.72 | 59.14 | 98.27 | 56.54 | 58.76 | 57.1 | 96.71 | 56.83 | 55.91 | 56.54 | 56.32 | 56.62 | 56.81 | 56.35 | 57.1 | 54.77 | 54.17 |
| EU979371.1/FADV-5/Australia | 63.12 | 64.29 | 65.61 | 64.12 | 59.47 | 99.83 | 67.44 | 66.78 | 63.95 | 64.29 | 63.29 | 64.29 | 63.95 | 59.8 | | 67.61 | 66.78 | 66.45 | 64.12 | 63.62 | 64.45 | 63.12 | 63.95 | 64.86 | 61.51 | 58.97 | 61.94 | 99.63 | 64.37 | 61.75 | 84.33 | 65.72 | 63.79 | 63.62 | 65.12 | 92.19 | 59.97 | 64.78 | 66.45 | 64.12 | 59.97 | 63.85 | 62.82 | 64.78 | 64.55 | 63.59 | 63.8 | 64.57 | 64.12 | 62.37 | 62.54 |
| EU979372.1/FADV-6/Australia | 62.92 | 81.54 | 70.81 | 69.8 | 57.93 | 67.44 | 99.83 | 85.91 | 81.04 | 70.97 | 63.09 | 71.48 | 69.63 | 58.6 | 67.61 | | 85.91 | 82.55 | 81.21 | 69.46 | 71.14 | 63.09 | 81.04 | 81.6 | 60.91 | 58.1 | 79.62 | 65.67 | 83.58 | 68.3 | 65.49 | 88.51 | 69.46 | 63.26 | 71.14 | 67.77 | 58.76 | 70.64 | 82.55 | 81.21 | 58.43 | 80.4 | 79.63 | 70.64 | 70.39 | 79.87 | 80.3 | 70.4 | 80.7 | 78.62 | 68.84 |
| EU979373.1/FADV-7/Australia | 63.26 | 88.42 | 69.3 | 67.95 | 58.6 | 66.94 | 85.74 | 100 | 88.09 | 68.96 | 63.42 | 69.63 | 67.79 | 59.1 | 66.78 | 85.91 | | 82.89 | 88.26 | 68.29 | 68.96 | 63.42 | 88.09 | 81.06 | 61.09 | 59.1 | 86.98 | 64.93 | 97.74 | 66.23 | 64.74 | 90.03 | 68.29 | 63.76 | 69.46 | 66.78 | 59.6 | 68.46 | 82.89 | 88.26 | 58.43 | 87.44 | 86.19 | 68.46 | 68.22 | 86.86 | 87.31 | 68.23 | 87.75 | 86.23 | 66.67 |
| EU979374.1/FADV-8a/Australia | 61.41 | 82.21 | 69.46 | 66.78 | 58.6 | 66.61 | 82.38 | 82.89 | 81.04 | 69.8 | 61.58 | 69.97 | 66.61 | 58.76 | 66.45 | 82.55 | 82.89 | | 81.21 | 66.11 | 69.8 | 61.58 | 81.04 | 99.45 | 59.09 | 59.1 | 79.06 | 64.18 | 80 | 64.72 | 63.62 | 83.11 | 66.11 | 61.74 | 70.47 | 66.94 | 59.27 | 69.13 | 100 | 81.21 | 58.76 | 82.08 | 81.32 | 69.13 | 68.89 | 81.53 | 81.97 | 68.9 | 82.38 | 79.89 | 67.75 |
| EU979375.1/FADV-8b/Australia | 61.07 | 97.48 | 68.29 | 68.12 | 56.59 | 64.29 | 81.04 | 88.26 | 99.83 | 67.95 | 61.24 | 68.12 | 67.95 | 57.1 | 64.12 | 81.21 | 88.26 | 81.21 | | 68.12 | 67.95 | 61.24 | 99.83 | 79.23 | 58.73 | 56.76 | 96.98 | 62.31 | 86.42 | 66.6 | 61.94 | 81.25 | 68.12 | 61.24 | 68.29 | 64.45 | 57.6 | 67.45 | 81.21 | 99.66 | 57.1 | 98.16 | 97.99 | 67.45 | 67.21 | 97.5 | 98 | 67.22 | 98.49 | 97.28 | 65.94 |
| EU979376.1/FADV-9/Australia | 61.24 | 67.79 | 73.86 | 94.1 | 56.21 | 63.62 | 69.3 | 68.29 | 68.12 | 76.39 | 61.41 | 76.05 | 94.27 | 56.54 | 63.62 | 69.46 | 68.29 | 66.11 | 68.12 | | 76.22 | 61.24 | 67.95 | 63.93 | 58.91 | 56.71 | 66.6 | 61.19 | 65.47 | 94.88 | 61.57 | 67.74 | 98.82 | 61.41 | 74.2 | 66.11 | 56.54 | 75.21 | 66.11 | 68.12 | 56.88 | 67.67 | 67.09 | 75.21 | 74.96 | 67.22 | 67.61 | 74.96 | 67.95 | 65.94 | 74.32 |
| EU979378.1/FADV-11/Australia | 60.07 | 67.95 | 94.1 | 75.38 | 56.21 | 64.29 | 70.97 | 68.96 | 67.95 | 99.83 | 60.23 | 98.99 | 75.21 | 56.38 | 64.45 | 71.14 | 68.96 | 69.8 | 67.95 | 76.22 | | 60.07 | 68.12 | 67.94 | 58 | 56.54 | 66.04 | 61.75 | 66.6 | 73.43 | 63.25 | 68.92 | 76.22 | 60.4 | 96.29 | 65.28 | 56.71 | 97.64 | 69.8 | 68.29 | 56.54 | 68.01 | 67.09 | 97.64 | 97.35 | 67.55 | 67.95 | 97.31 | 68.29 | 65.94 | 99.27 |
| GU952110.1/FADV-1/Poland | 99.46 | 65.18 | 62.48 | 64.24 | 65.04 | 66.13 | 66.26 | 67.34 | 65.45 | 63.29 | 99.49 | 59.73 | 61.24 | 62.9 | 63.12 | 63.09 | 63.42 | 61.58 | 61.24 | 61.24 | 60.07 | | 65.45 | 59.38 | 99.63 | 63.88 | 59.45 | 62.41 | 61.86 | 59.62 | 60.37 | 64.17 | 63.62 | 98.64 | 62.21 | 65.86 | 65.58 | 62.75 | 65.59 | 65.59 | 65.99 | 64.16 | 63.84 | 62.89 | 62.7 | 64.74 | 65.19 | 62.72 | 64.24 | 56.62 | 53.31 |
| JN112373.1/FADV-8b/Canada | 66.75 | 97.9 | 71.57 | 71.45 | 61.7 | 69.69 | 84.05 | 90.23 | 99.38 | 71.57 | 61.07 | 68.29 | 67.79 | 56.93 | 63.95 | 81.04 | 88.09 | 81.04 | 99.83 | 67.95 | 68.12 | 65.45 | | 79.05 | 58.55 | 59.97 | 97.13 | 65.46 | 86.62 | 67.6 | 64.17 | 83.06 | 70.89 | 66.96 | 71.69 | 69.69 | 62.44 | 70.95 | 84.92 | 99.75 | 62.07 | 97.79 | 98.04 | 70.14 | 70.12 | 97.42 | 97.87 | 70.09 | 91.23 | 85.54 | 56.77 |
| KC750785.1/FADV-8a/Hungry | 59.2 | 80.33 | 67.58 | 65.03 | 56.7 | 64.86 | 81.42 | 81.06 | 79.05 | 67.94 | 59.38 | 68.12 | 65.03 | 56.88 | 64.86 | 81.6 | 81.06 | 99.45 | 79.23 | 63.93 | 67.94 | 59.38 | 79.05 | | 59.38 | 57.25 | 77.71 | 63.35 | 79.49 | 64.1 | 62.96 | 81.6 | 63.93 | 59.74 | 68.67 | 65.05 | 57.43 | 67.21 | 99.45 | 79.23 | 56.88 | 80.51 | 80.05 | 67.21 | 66.95 | 79.93 | 80.22 | 66.97 | 80.51 | 79.21 | 66.92 |
| KC750802.1/FADV-1/Hungry | 99.63 | 58.36 | 57.09 | 58.91 | 60.88 | 61.51 | 60.73 | 61.09 | 58.73 | 58 | 99.82 | 57.64 | 58.91 | 61.24 | 61.51 | 60.91 | 61.09 | 59.09 | 58.73 | 58.91 | 58 | 99.63 | 58.55 | 59.38 | | 61.06 | 56.02 | 60.23 | 59.96 | 57.99 | 58.09 | 59.09 | 59.09 | 98.71 | 57.09 | 60.79 | 61.79 | 57.45 | 59.09 | 58.73 | 62.16 | 58.73 | 58.56 | 57.45 | 57.21 | 58.3 | 58.51 | 57.25 | 58.73 | 58.22 | 56.9 |
| KM217572.1/FADV-4/Pakistan | 61.4 | 57.09 | 57.26 | 56.05 | 92.29 | 59.2 | 58.56 | 59.22 | 57.22 | 56.72 | 62.9 | 56.71 | 56.54 | 97.06 | 58.97 | 58.1 | 59.1 | 59.1 | 56.76 | 56.71 | 56.54 | 63.88 | 59.97 | 57.25 | 61.06 | | 58.41 | 60.93 | 60.44 | 57.4 | 58.63 | 58.82 | 59.35 | 64.81 | 60.99 | 62.59 | 96.8 | 60.14 | 62.71 | 60.59 | 96.36 | 57.1 | 55.35 | 58.78 | 59.69 | 59.61 | 58.93 | 59.62 | 53.11 | 48.64 | 46.6 |
| KY426984.1/FADV-8b/China | 57.67 | 92.34 | 64.75 | 64.31 | 54.77 | 62.28 | 75.99 | 83.21 | 91.61 | 63.86 | 57.36 | 66.6 | 66.42 | 53.85 | 61.94 | 79.62 | 86.98 | 79.06 | 96.98 | 66.6 | 66.04 | 59.45 | 97.13 | 77.71 | 56.02 | 58.41 | | 66.05 | 87.48 | 67.93 | 63.68 | 79.47 | 68.09 | 61.66 | 68.86 | 65.89 | 59.19 | 67.92 | 81.38 | 97.03 | 59.19 | 95.16 | 95.57 | 66.09 | 67.03 | 94.88 | 94.24 | 67.1 | 86.07 | 82.16 | 55.71 |
| LC604661.1/FADV-5/Japan | 60.67 | 62.34 | 61.99 | 60.38 | 58.04 | 93.71 | 65.11 | 64.23 | 62.34 | 61.11 | 61.19 | 61.57 | 61.57 | 57.46 | 99.63 | 65.67 | 64.93 | 64.18 | 62.31 | 61.19 | 61.75 | 62.41 | 65.46 | 63.35 | 60.23 | 60.93 | 66.05 | | 67.44 | 63.52 | 85.99 | 63.53 | 63.19 | 64.34 | 65.43 | 91.94 | 61.86 | 64.96 | 67.91 | 66.2 | 61.86 | 62.59 | 60.6 | 63.16 | 64.14 | 64.92 | 64.2 | 64.22 | 57.52 | 54.57 | 52.87 |
| MH186135.1/FADV-7/China | 59.73 | 82.18 | 64.6 | 63.86 | 56.68 | 63.45 | 78.94 | 91.31 | 82.18 | 64.31 | 60.57 | 66.98 | 65.47 | 56.47 | 64.37 | 83.58 | 97.74 | 80 | 86.42 | 65.47 | 66.6 | 61.86 | 86.62 | 79.49 | 59.96 | 60.44 | 87.48 | 67.44 | | 67.27 | 65.96 | 88.59 | 67.43 | 64.16 | 69.33 | 66.82 | 60.9 | 68.23 | 81.85 | 87.01 | 60.12 | 85.3 | 84.88 | 66.25 | 67.19 | 85.25 | 84.58 | 67.26 | 77.31 | 74.18 | 56.18 |
| ON502505.1/FADV-3/China | 60.86 | 67.27 | 71.9 | 98.68 | 56.91 | 63.36 | 69.24 | 67.76 | 67.76 | 74.88 | 59.43 | 72.87 | 98.67 | 54.72 | 61.75 | 68.3 | 66.23 | 64.72 | 66.6 | 94.88 | 73.43 | 59.62 | 67.6 | 64.1 | 57.99 | 57.4 | 67.93 | 63.52 | 67.27 | | 63.84 | 66.16 | 94.88 | 60.69 | 72.89 | 64.82 | 57.24 | 73.39 | 66.28 | 67.6 | 57.89 | 66.13 | 65.44 | 73.39 | 73.14 | 67.05 | 67.27 | 73.15 | 62.99 | 60.53 | 64.63 |
| ON502563.1/FADV-5/China | 61.56 | 63.36 | 65.64 | 63.68 | 58.96 | 85.83 | 67.43 | 66.94 | 64.5 | 65.31 | 59.14 | 63.81 | 61.75 | 56.34 | 84.33 | 65.49 | 64.74 | 63.62 | 61.94 | 61.57 | 63.25 | 60.37 | 64.17 | 62.96 | 58.09 | 58.63 | 63.68 | 85.99 | 65.96 | 63.84 | | 62.59 | 63.36 | 62.05 | 65.96 | 86.16 | 59.28 | 65.31 | 66.29 | 64.33 | 60.42 | 62.23 | 60.18 | 65.31 | 65.08 | 63.97 | 64.18 | 65.1 | 59.77 | 56.84 | 56.03 |
| ON502566.1/FADV-6/China | 64.02 | 83.51 | 70.31 | 70.01 | 59.7 | 68.05 | 89.36 | 91 | 82.91 | 70.76 | 61.49 | 69.26 | 67.91 | 58.15 | 65.72 | 88.51 | 90.03 | 83.11 | 81.25 | 67.74 | 68.92 | 64.17 | 83.06 | 81.6 | 59.09 | 58.82 | 79.47 | 63.53 | 88.59 | 66.16 | 62.59 | | 69.72 | 64.47 | 71.36 | 68.05 | 60.3 | 70.16 | 84.86 | 83.21 | 60 | 82.49 | 80.83 | 70.16 | 69.95 | 81.49 | 82.24 | 69.96 | 82.61 | 78.83 | 66.61 |
| ON502596.1/FADV-9/China | 64.49 | 70.76 | 76.68 | 94.5 | 60.88 | 66.75 | 71.83 | 71.16 | 71.03 | 79.22 | 61.58 | 76.05 | 94.1 | 56.71 | 63.79 | 69.46 | 68.29 | 66.11 | 68.12 | 98.82 | 76.22 | 63.62 | 70.89 | 63.93 | 59.09 | 59.35 | 68.09 | 63.19 | 67.43 | 94.88 | 63.36 | 69.72 | | 64.62 | 76.94 | 68.74 | 60.88 | 78.42 | 69.43 | 70.89 | 61.28 | 69.67 | 69.5 | 78.42 | 78.21 | 70.01 | 70.61 | 78.21 | 67.16 | 60.95 | 66.03 |
| PV457637.1/FADV-1/Korea | 94.37 | 63.56 | 61.66 | 62.82 | 63.63 | 64.93 | 64.38 | 66.01 | 63.91 | 62.24 | 98.81 | 60.07 | 61.41 | 63.41 | 63.62 | 63.26 | 63.76 | 61.74 | 61.24 | 61.41 | 60.4 | 98.64 | 66.96 | 59.74 | 98.71 | 64.81 | 61.66 | 64.34 | 64.16 | 60.69 | 62.05 | 64.47 | 64.62 | | 64.35 | 67.88 | 66.91 | 64.84 | 67.64 | 67.03 | 67.52 | 64.14 | 63.93 | 63.16 | 63.78 | 65.51 | 65.43 | 63.75 | 59.7 | 52.04 | 49.02 |
| PV457640.1/FADV-2/Korea | 61 | 68.72 | 91.47 | 73.6 | 60.07 | 65.78 | 70.58 | 69.3 | 68.6 | 91.94 | 59.4 | 95.95 | 73.19 | 57.72 | 65.12 | 71.14 | 69.46 | 70.47 | 68.29 | 74.2 | 96.29 | 62.21 | 71.69 | 68.67 | 57.09 | 60.99 | 68.86 | 65.43 | 69.33 | 72.89 | 65.96 | 71.36 | 76.94 | 64.35 | | 70.17 | 63.14 | 96.46 | 73.97 | 72.02 | 63.14 | 70.76 | 70.45 | 94.54 | 95.96 | 70.55 | 70.37 | 95.93 | 65.64 | 58.4 | 81.91 |
| PV457652.1/FADV-5/Korea | 64.62 | 66.97 | 67.05 | 66.24 | 60.92 | 89.13 | 68.71 | 68.36 | 66.86 | 66.71 | 62.62 | 65.45 | 65.61 | 59.14 | 92.19 | 67.77 | 66.78 | 66.94 | 64.45 | 66.11 | 65.28 | 65.86 | 69.69 | 65.05 | 60.79 | 62.59 | 65.89 | 91.94 | 66.82 | 64.82 | 86.16 | 68.05 | 68.74 | 67.88 | 70.17 | | 63.89 | 69.81 | 72.22 | 70.29 | 64.73 | 67.44 | 67.21 | 67.85 | 68.84 | 68.57 | 68.26 | 68.81 | 62.94 | 55.32 | 54.12 |
| PV457659.1/FADV-4/Korea | 63.79 | 59.79 | 59.95 | 58.67 | 93.58 | 61.5 | 60.83 | 61.53 | 59.79 | 59.25 | 63.74 | 56.88 | 56.38 | 98.27 | 59.97 | 58.76 | 59.6 | 59.27 | 57.6 | 56.54 | 56.71 | 65.58 | 62.44 | 57.43 | 61.79 | 96.8 | 59.19 | 61.86 | 60.9 | 57.24 | 59.28 | 60.3 | 60.88 | 66.91 | 63.14 | 63.89 | | 62.41 | 64.36 | 63.03 | 96.14 | 60.03 | 59.09 | 60.94 | 61.88 | 61.72 | 61.38 | 61.72 | 56.16 | 49.4 | 46.75 |
| PV457662.1/FADV-11/Korea | 61.58 | 68.26 | 91.24 | 74.18 | 59.37 | 65.55 | 70.12 | 68.6 | 67.91 | 92.87 | 59.73 | 96.96 | 73.69 | 56.54 | 64.78 | 70.64 | 68.46 | 69.13 | 67.45 | 75.21 | 97.64 | 62.75 | 70.95 | 67.21 | 57.45 | 60.14 | 67.92 | 64.96 | 68.23 | 73.39 | 65.31 | 70.16 | 78.42 | 64.84 | 96.46 | 69.81 | 62.41 | | 72.99 | 71.29 | 62.17 | 69.93 | 69.68 | 98.14 | 99.65 | 69.69 | 69.63 | 99.62 | 64.89 | 57.64 | 83.13 |
| PV457664.1/FADV-8a/Korea | 64.14 | 82.09 | 69.73 | 67.75 | 60.9 | 68.67 | 81.74 | 82.91 | 81.28 | 70.2 | 61.58 | 69.97 | 66.61 | 58.76 | 66.45 | 82.55 | 82.89 | 100 | 81.21 | 66.11 | 69.8 | 65.59 | 84.92 | 99.45 | 59.09 | 62.71 | 81.38 | 67.91 | 81.85 | 66.28 | 66.29 | 84.86 | 69.43 | 67.64 | 73.97 | 72.22 | 64.36 | 72.99 | | 85.28 | 64 | 84.69 | 84.48 | 70.95 | 71.89 | 84.43 | 84.32 | 71.86 | 78.37 | 69.89 | 57.49 |
| PV457669.1/FADV-8b/Korea | 63.91 | 93.49 | 68.68 | 68.34 | 59.4 | 67.05 | 80.35 | 86.28 | 94.65 | 68.68 | 61.24 | 68.46 | 67.95 | 57.1 | 64.12 | 81.21 | 88.26 | 81.21 | 99.66 | 68.12 | 68.29 | 65.59 | 99.75 | 79.23 | 58.73 | 60.59 | 97.03 | 66.2 | 87.01 | 67.6 | 64.33 | 83.21 | 70.89 | 67.03 | 72.02 | 70.29 | 63.03 | 71.29 | 85.28 | | 62.67 | 97.66 | 97.88 | 69.34 | 70.25 | 96.71 | 96.67 | 70.22 | 89.99 | 83.96 | 56.28 |
| PV457670.1/FADV-10/Korea | 64.37 | 59.44 | 59.6 | 59.25 | 92.75 | 61.73 | 60.6 | 60.72 | 59.56 | 59.14 | 64.25 | 56.71 | 56.88 | 96.71 | 59.97 | 58.43 | 58.43 | 58.76 | 57.1 | 56.88 | 56.54 | 65.99 | 62.07 | 56.88 | 62.16 | 96.36 | 59.19 | 61.86 | 60.12 | 57.89 | 60.42 | 60 | 61.28 | 67.52 | 63.14 | 64.73 | 96.14 | 62.17 | 64 | 62.67 | | 59.34 | 58.32 | 60.69 | 61.63 | 61.23 | 60.89 | 61.47 | 55.54 | 48.95 | 46.75 |
| PX401873/FADV-8b/NIS-1/Malaysia | 64 | 96.14 | 70.62 | 70.07 | 59.07 | 67.31 | 82.9 | 88.83 | 97.66 | 70.48 | 60.97 | 68.17 | 67.5 | 56.83 | 63.85 | 80.4 | 87.44 | 82.08 | 98.16 | 67.67 | 68.01 | 64.16 | 97.79 | 80.51 | 58.73 | 57.1 | 95.16 | 62.59 | 85.3 | 66.13 | 62.23 | 82.49 | 69.67 | 64.14 | 70.76 | 67.44 | 60.03 | 69.93 | 84.69 | 97.66 | 59.34 | | 99.72 | 69.79 | 69.59 | 98.26 | 99.04 | 69.6 | 99.31 | 95.33 | 63.56 |
| PX401874/FADV-8b/Yan-2/Malaysia | 63.67 | 96.49 | 70.45 | 70.3 | 58.17 | 66.76 | 82.94 | 88.17 | 97.88 | 70.3 | 60.16 | 67.28 | 67.09 | 55.91 | 62.82 | 79.63 | 86.19 | 81.32 | 97.99 | 67.09 | 67.09 | 63.84 | 98.04 | 80.05 | 58.56 | 55.35 | 95.57 | 60.6 | 84.88 | 65.44 | 60.18 | 80.83 | 69.5 | 63.93 | 70.45 | 67.21 | 59.09 | 69.68 | 84.48 | 97.88 | 58.32 | 99.72 | | 69.27 | 69.14 | 98.98 | 99.57 | 69.05 | 99.57 | 99.03 | 64.74 |
| PX401875/FADV-11/Yan-3/Malaysia | 60.09 | 66.39 | 89.57 | 72.75 | 57.97 | 63.77 | 68.4 | 66.75 | 66.04 | 91.23 | 59.73 | 96.96 | 73.69 | 56.54 | 64.78 | 70.64 | 68.46 | 69.13 | 67.45 | 75.21 | 97.64 | 62.89 | 70.14 | 67.21 | 57.45 | 58.78 | 66.09 | 63.16 | 66.25 | 73.39 | 65.31 | 70.16 | 78.42 | 63.16 | 94.54 | 67.85 | 60.94 | 98.14 | 70.95 | 69.34 | 60.69 | 69.79 | 69.27 | | 99.78 | 69.2 | 69.94 | 99.75 | 65.5 | 58.19 | 83.72 |
| PX401876/FADV-11/Yan-5/Malaysia | 63.53 | 70.5 | 95.2 | 77.13 | 61.63 | 67.7 | 72.78 | 70.88 | 70.12 | 96.98 | 59.51 | 96.68 | 73.44 | 56.32 | 64.55 | 70.39 | 68.22 | 68.89 | 67.21 | 74.96 | 97.35 | 62.7 | 70.12 | 66.95 | 57.21 | 59.69 | 67.03 | 64.14 | 67.19 | 73.14 | 65.08 | 69.95 | 78.21 | 63.78 | 95.96 | 68.84 | 61.88 | 99.65 | 71.89 | 70.25 | 61.63 | 69.59 | 69.14 | 99.78 | | 69.55 | 70.12 | 99.52 | 66.28 | 59.26 | 85.44 |
| PX401877/FADV-8b/Yan-6/Malaysia | 62.4 | 90.9 | 67.2 | 66.85 | 58.21 | 65.45 | 78.48 | 84.34 | 92.3 | 66.97 | 60.73 | 67.72 | 67.22 | 56.62 | 63.59 | 79.87 | 86.86 | 81.53 | 97.5 | 67.22 | 67.55 | 64.74 | 97.42 | 79.93 | 58.3 | 59.61 | 94.88 | 64.92 | 85.25 | 67.05 | 63.97 | 81.49 | 70.01 | 65.51 | 70.55 | 68.57 | 61.72 | 69.69 | 84.43 | 96.71 | 61.23 | 98.26 | 98.98 | 69.2 | 69.55 | | 98.55 | 69.59 | 90.91 | 85.52 | 55.72 |
| PX401878/FADV-8b/Yan-7/Malaysia | 62.31 | 90.82 | 67.02 | 66.55 | 57.86 | 65.15 | 78.35 | 84.24 | 92.24 | 66.9 | 60.93 | 68.11 | 67.45 | 56.81 | 63.8 | 80.3 | 87.31 | 81.97 | 98 | 67.61 | 67.95 | 65.19 | 97.87 | 80.22 | 58.51 | 58.93 | 94.24 | 64.2 | 84.58 | 67.27 | 64.18 | 82.24 | 70.61 | 65.43 | 70.37 | 68.26 | 61.38 | 69.63 | 84.32 | 96.67 | 60.89 | 99.04 | 99.57 | 69.94 | 70.12 | 98.55 | | 70.16 | 91.8 | 86.04 | 56.06 |
| PX401879/FADV-11/Yan-8/Malaysia | 63.5 | 70.47 | 95.17 | 77.1 | 61.47 | 67.67 | 72.75 | 70.85 | 70.09 | 96.95 | 59.53 | 96.64 | 73.45 | 56.35 | 64.57 | 70.4 | 68.23 | 68.9 | 67.22 | 74.96 | 97.31 | 62.72 | 70.09 | 66.97 | 57.25 | 59.62 | 67.1 | 64.22 | 67.26 | 73.15 | 65.1 | 69.96 | 78.21 | 63.75 | 95.93 | 68.81 | 61.72 | 99.62 | 71.86 | 70.22 | 61.47 | 69.6 | 69.05 | 99.75 | 99.52 | 69.59 | 70.16 | | 66.29 | 59.19 | 85.29 |
| PX401880/FADV-8b/Kon-1/Malaysia | 56.84 | 84.57 | 62.5 | 61.91 | 52.76 | 59.95 | 73.03 | 78.33 | 85.87 | 62.5 | 61.24 | 68.46 | 67.79 | 57.1 | 64.12 | 80.7 | 87.75 | 82.38 | 98.49 | 67.95 | 68.29 | 64.24 | 91.23 | 80.51 | 58.73 | 53.11 | 86.07 | 57.52 | 77.31 | 62.99 | 59.77 | 82.61 | 67.16 | 59.7 | 65.64 | 62.94 | 56.16 | 64.89 | 78.37 | 89.99 | 55.54 | 99.31 | 99.57 | 65.5 | 66.28 | 90.91 | 91.8 | 66.29 | | 94.01 | 61.73 |
| PX401881/FADV-8b/Yan-10/Malaysia | 48.71 | 77.89 | 55.14 | 54.71 | 45.8 | 52.41 | 64.62 | 71.04 | 79.6 | 54.43 | 58.88 | 66.12 | 65.94 | 54.77 | 62.37 | 78.62 | 86.23 | 79.89 | 97.28 | 65.94 | 65.94 | 56.62 | 85.54 | 79.21 | 58.22 | 48.64 | 82.16 | 54.57 | 74.18 | 60.53 | 56.84 | 78.83 | 60.95 | 52.04 | 58.4 | 55.32 | 49.4 | 57.64 | 69.89 | 83.96 | 48.95 | 95.33 | 99.03 | 58.19 | 59.26 | 85.52 | 86.04 | 59.19 | 94.01 | | 60.87 |
| PX401882/FADV-11/Kon-5/Malaysia | 46.29 | 52.78 | 75.04 | 58.82 | 43.71 | 50.57 | 55.21 | 53.35 | 52.78 | 79.05 | 57.97 | 98.18 | 73.04 | 54.17 | 62.54 | 68.84 | 66.67 | 67.75 | 65.94 | 74.32 | 99.27 | 53.31 | 56.77 | 66.92 | 56.9 | 46.6 | 55.71 | 52.87 | 56.18 | 64.63 | 56.03 | 66.61 | 66.03 | 49.02 | 81.91 | 54.12 | 46.75 | 83.13 | 57.49 | 56.28 | 46.75 | 63.56 | 64.74 | 83.72 | 85.44 | 55.72 | 56.06 | 85.29 | 61.73 | 60.87 | |

**Supplementary Figure S3:** Nucleotide sequence alignment of partial hexon gene sequences from 51 selected FAdV sequences, including the ten newly identified isolates from this study, was performed in Geneious Prime (version 2025.1.1) using MAFFT (version 7.450) with the G-INS-i algorithm, applying a gap opening penalty of 1.53 and an offset value of 0.123. The alignment revealed sequence homology ranging from 43.71% to 99.83%.
